# DNA Methylation in Gastric Cancer and Preneoplastic Lesions: Emerging Insights and Future Directions

**DOI:** 10.3390/cancers18071075

**Published:** 2026-03-26

**Authors:** Carlotta Ceccon, Giulia Maddalena, Valentina Angerilli, Floriana Nappo, Jessica Gasparello, Marianna Sabbadin, Luisa Toffolatti, Francesca Bergamo, Matteo Fassan, Sara Lonardi

**Affiliations:** 1 Department of Surgery, Oncology and Gastroenterology, University of Padua, 35128 Padua, Italy; 2 Surgical Pathology Unit, ULSS2 Marca Trevigiana, 31100 Treviso, Italymatteo.fassan@unipd.it (M.F.); 3 Veneto Institute of Oncology IOV-IRCCS, 35128 Padua, Italy; 4 Department of Medicine, Surgical Pathology Unit, University of Padua, 35128 Padua, Italy

**Keywords:** gastric cancer, precancerous gastric lesions, DNA methylation, epigenetic biomarkers, target therapy, precision oncology

## Abstract

Gastric cancer (GC) represents one of the most common and lethal cancers worldwide. The lack of early detection methods and wide heterogeneity from both histopathological and molecular points of view contribute to the high mortality associated with GC. Nowadays, the growing interest in the epigenetic landscape appears to represent a promising approach for improving early diagnosis, disease monitoring, therapy selection, and drug response. The knowledge of the epigenetic landscape in cancerous and, especially in precancerous lesions, remain scarce and controversial. The limitations must be identified due to the lack of standardization in experimental settings which introduce bias and lead to discrepancies in final conclusions. This review aims to provide an overview of epigenetics in the diagnostic setting of gastric cancer and in preneoplastic conditions, illustrating the available experimental approaches and therapeutic management in the era of epigenetics.

## Simple Summary

DNA methylation is a key epigenetic mechanism that regulates gene expression without altering the DNA sequence. In gastric cancer (GC), abnormal methylation—in cooperation with other molecular and epigenetic mechanisms—promotes tumor development by silencing tumor suppressor genes or activating oncogenes. Various biomarkers have been tested and could be promising for improving GC molecular subtypes and predicting therapy responses. Despite their clinical potential, nowadays, robust standardization and further validation are needed before implementation in routine practice.

## 1. Background

Gastric cancer (GC) is the fifth most diagnosed cancer worldwide, twice as high in males as in females. According to the most recent GLOBOCAN estimates, GC was responsible for 968,000 new cases in 2022 and ranks as the third cause of cancer-related mortality, leading to 660,000 deaths. Despite a steady decline in mortality and incidence over the last 15 years, mainly attributable to a decrease in risk factors and advances in surgical and systemic therapies, prognosis remains dismal, with a 5-year survival rate under 30% in most countries [1]. The low survival rate of GC is largely due to late diagnosis and the lack of effective early detection strategies, compounded by the high heterogeneity of the disease from both morphological and molecular standpoints, which also makes proper and unified classification of the disease challenging [2,3]. Accumulating evidence indicates that epigenetic alterations, in cooperation with genetic events, play a crucial role in gastric cancer development and progression [4].

Of particular note is the link between DNA methylation and GC initiation and evolution. This phenomenon has been well described in various cancer types, such as endometrial and colorectal cancer, highlighting its key role in prognosis and the success of immunotherapy with checkpoint inhibitors (ICIs) [5]. To date, while the understanding of therapeutic implications of DNA methylation in gastric cancer remains limited, methylation changes have been identified in premalignant lesions. This highlights their potential as early biomarkers of carcinogenesis, as they can be detected before the onset of overt carcinoma. This review aims to describe the current understanding of methylation in preneoplastic lesions and gastric cancer carcinogenesis, highlighting the biomarker’s utility for clinical scenarios. We report the different technical approaches for its detection and analysis and explore possible future applications, giving a comprehensive translational overview particularly valuable for clinical oncologists, as it bridges the gap between epigenetic mechanisms and potential diagnostic utility.

## 2. The Role of DNA Methylation in Epigenetics and Carcinogenesis

Methylation is an important type of epigenetic regulation mediated by DNA methyltransferases (DNMTs), which are able to transfer an active methyl (CH_3_) group onto the C5 position of the cytosine to form 5-methylcytosine (m5C) without altering DNA sequence composition [6]. DNMT1, the most abundant maintenance DNMT in mammalian tissues, copies existing methylation patterns during DNA replication by targeting hemimethylated DNA. DNMT3a and DNMT3b are de novo methyltransferases that establish new methylation marks, with DNMT3a expressed ubiquitously and DNMT3b mainly during early development and in specific tissues. DNMT3L, although catalytically inactive, enhances the activity of DNMT3a/b. Methylation can occur at different levels in DNA, RNA, histone, and non-histone proteins, playing a broad role in the reversible control of cell behavior. DNA methylation regulates gene expression at the transcriptional level; RNA methylation primarily influences RNA processing and degradation; and protein methylation impacts protein function, guiding translation, localization, and signaling pathways [6,7,8,9].

DNA methylation preferentially occurs (98%) in -C- phosphor -G- site (CpG) island loci, which often cluster into CpG islands (~1000 bp, high CpG density) found in approximately 70% of gene promoters, especially of tumor suppressor genes. Normally, promoter CpG islands are unmethylated, allowing an open chromatin structure and transcription factor binding. Hypermethylation of these islands leads to stable, heritable gene silencing, a hallmark of cancers such as the CpG island methylator phenotype (CIMP) in gastric cancer. Gene silencing occurs via two main mechanisms: (1) direct interference, where methyl groups block transcription factor binding, and (2) recruitment of repressive proteins, where methyl-binding domain (MBD) proteins (e.g., MeCP2, MBD1, MBD2) attract co-repressor complexes, including histone deacetylases (HDACs) and histone methyltransferases (HMTs), condensing DNA into transcriptionally inactive heterochromatin [10]. Methylation regulates gene expression and function both in physiologic processes and carcinogenesis through gene promoter up- and down-regulation. Cancer-associated methylation changes generally involve two distinct and functionally different processes. Promoter CpG island hypermethylation leads to transcriptional silencing of tumor suppressor genes through the repression of promoter activity and interference with transcription factor binding. In contrast, genome-wide DNA hypomethylation, frequently affecting repetitive DNA elements, is strongly associated with genomic instability. Loss of methylation in these regions can lead to oncogene activation, increased mutation rates at the DNA level, aneuploidies, and chromosomal fragility. In 1983, Feinberg and Vogelstein described that cancer tissues exhibit global DNA hypomethylation compared with their normal counterparts, highlighting one of the earliest recognized epigenetic alterations in tumorigenesis. Subsequent studies showed that, in addition to global hypomethylation, specific CpG islands and the nearby CpG island shores (the regions within 2 kb of the CpG islands) can undergo aberrant methylation in neoplastic cells, leading to a dysregulated expression of key tumor suppressor genes (TSGs) [11,12,13]. Accumulating evidence suggests that these methylation alterations arise early during tumor development. Indeed, both benign polyps and malignant tissues display global DNA hypomethylation compared with normal mucosa, supporting a potential role for this epigenetic alteration in the earliest stages of carcinogenesis [14].

In 1997, Stirzaker and colleagues first described the role of methylation as a driver of oncogenesis in the retinoblastoma gene (*RB*) promoter in affected patients [15]. *RB* methylation is one of the strongest evidence highlighting the causal role of aberrant methylation of CpG islands in the neoplastic setting. The *RB* is commonly active in precursor tumor cells, and promoter methylation results in the same effect as a genetic mutation, leading to *RB* downregulation.

Sporadic microsatellite unstable colorectal cancer (CRC), accounting for approximately 15% of all cases, represents another tumor type in which aberrant DNA methylation plays a key role. These tumors are characterized by microsatellite instability (MSI), due to epigenetic silencing of the *MLH1* gene through promoter methylation (p*MLH1*), rather than germline mutations in mismatch repair (MMR) genes [14,16]. This mechanism promotes a hyperproliferative phenotype in early carcinogenesis, highlighting aberrant DNA methylation as a driver of tumor development and progression.

Through the years, other TSGs have been identified in different types of tumors, and together with the presence of methylation appearing before mutations, are strong evidence supporting this theory [17].

DNA hypermethylation is a valuable diagnostic and prognostic biomarker, as it affects specific genes at different stages of tumor development, enabling discrimination between premalignant lesions and advanced disease. The CpG island methylator phenotype, first described in CRC, defines a subgroup characterized by high promoter methylation and poor prognosis (19). Moreover, hypermethylation-mediated silencing of genes such as CDH1 (E-cadherin), SOX11, and DNA repair-related genes, including MLH1, contributes to tumor progression, genomic instability, and altered therapeutic sensitivity across multiple cancer types, including colorectal, gastric, prostate, and endometrial cancers [12,18,19].

In the era of precision medicine, due to its reversibility and dynamism, epigenetic changes represent an attractive diagnostic, prognostic and therapeutic target across several diseases, including cancer. DNA methylation represents a valuable tool for disease detection, monitoring therapy outcome and progression. Indeed, accumulating evidence indicates that a dysregulated DNA methylation pattern can predict patients’ response to chemo- and immunotherapy, leading to identification of tumor-specific drug-responsive biomarkers [20]. For example, O(6)-methylguanine DNA methyltransferase (*MGMT*), a DNA repair enzyme responsible for removing a methyl-group from O(6)-alkylguanine, is silenced through promoter methylation. This epigenetic alteration is a positive predictor of response to alkylating agents and is associated with improved both overall survival (OS) and progression-free survival (PFS) [21]. To date, two methylation-based biomarkers—SEPT9 in combination with NDRG4 and BMP3—have been approved by the Food and Drug Administration (FDA) for CRC screening [22]. Additionally, several studies demonstrate that the *CHFR* gene methylation, which plays a key role in G2/M cell-cycle checkpoint and chromosomal integrity, has emerged as a predictive biomarker for taxanes sensitivity in several tumor types, including esophageal, gastric, cervical, lung, endometrial cancers, and oral squamous cell carcinoma. Notably, a longer OS has been observed in docetaxel-treated GC patients with *CHFR* methylated, compared to the unmethylated group [20,23].

## 3. Gastric Cancer Progression: An Overview of Molecular Alterations and the Role of DNA Methylation

In 1975, Pelayo Correa first proposed a stepwise model of gastric carcinogenesis. This multistep cascade significantly improved the understanding of gastric tumorigenesis and contributed to the development of effective preventive strategies for gastric cancer (GC) [24]. The sequence begins with *Helicobacter pylori*-induced chronic gastritis, which may progress to chronic atrophic gastritis, characterized by the loss of normal gastric glands. The most common form of atrophic gastritis is intestinal metaplasia (IM), in which the gastric epithelium is substituted by the intestinal epithelium. The process may then advance to gastric dysplasia, classified as low- or high-grade, representing neoplastic lesions confined to the epithelium. The final step of this cascade is the development of invasive gastric carcinoma, which occurs after neoplastic cells acquire stromal invasive capability [25].

According to the Laurén classification, gastric cancer is broadly divided into intestinal-type and diffuse-type tumors. The intestinal type typically develops through the Correa cascade described above and is strongly associated with environmental factors, including *Helicobacter pylori* infection. In contrast, diffuse-type gastric cancer generally does not arise from well-defined preneoplastic lesions and is more frequently associated with genetic alterations, such as *CDH1* mutations. These two subtypes also display distinct molecular and epigenetic features, including differences in DNA methylation profiles [2].

From a histopathological standpoint, the carcinogenesis process has been largely validated by both large epidemiological and longitudinal studies, even if the knowledge of molecular mechanisms driving each transition is still scarce. To date, the putative role of *Helicobacter pylori* infection in the pathogenesis of chronic gastritis has been well established, contributing to persistent inflammation, oxidative DNA damage, and the emergence of early somatic mutations, particularly in the *TP53* and *ARID1A* genes [26]. In the progression to IM, additional genetic alterations emerge, including chromosomal gains (e.g., 8q), low-frequency mutations, and telomere shortening [27]. Dysplasia is characterized by an increased mutational burden and frequent alterations in key tumor suppressor genes such as *APC*, *TP53*, and *KRAS* [28]. In the final phase of carcinogenesis, these and additional molecular alterations converge to promote uncontrolled cell proliferation, deregulation of key signaling pathways, and the acquisition of invasive and metastatic properties [29].

Among the molecular alterations involved in this stepwise cascade, epigenetic modifications, particularly the DNA methylation profile, may enhance the understanding of tumor-intrinsic heterogeneity and provide a molecular framework for elucidating the pathogenesis of individual cases [30]. Unlike genetic mutations, epigenetic modifications are potentially reversible, positioning them as promising candidates for early therapeutic strategies. Notably, increasing evidence indicates that alterations in DNA methylation can be observed not only in established carcinomas but also in premalignant lesions, and may even precede detectable histopathological changes [29,31].

Among epigenetic alterations, aberrant DNA methylation is especially closely linked to the oncogenesis and progression of GC. In this context, gastric cancer development can be accelerated by hypermethylation across multiple gene promoters, leading to the previously described CpG island methylator phenotype. Likewise, for colorectal cancer, and also for GC, the CIMP status discriminates gastric neoplasia in different subgroups. However, the precise definition and biological significance of CIMP in GC remain controversial and not universally standardized across studies. For example, in colorectal cancer, the CIMP has been more consistently characterized, but in GC, variability exists in how the CIMP is defined and measured, leading to differences in reported associations [18,32,33]. Nevertheless, in 2014, The Cancer Genome Atlas (TCGA) project analyzed 295 primary gastric adenocarcinoma tumor tissues, setting up a molecular classification based on the genomic landscape, dividing GCs into four groups: positive for Epstein–Barr virus (EBV), tumors with microsatellite instability (MSI), genomically stable tumors (GS), and tumors with chromosomal instability (CIN) [34,35]. These classifications provide a framework for understanding GC heterogeneity, while the role of the CIMP remains an emerging and incompletely defined layer of epigenetic stratification in gastric cancer.

In the molecular classification of gastric adenocarcinoma, DNA methylation patterns serve as a primary distinguishing feature, particularly for EBV-positive and MSI subtypes. EBV-positive tumors exhibit extreme DNA hypermethylation, referred to as EBV-CIMP, and show the highest prevalence of methylation reported across cancer types, with universal silencing of the *CDKN2A* (p16INK4A) gene and typically no hypermethylation of the *MLH1* promoter. MSI tumors also display high levels of hypermethylation, termed Gastric-CIMP, but with a profile distinct from EBV-CIMP; their hallmark is *MLH1* silencing through promoter hypermethylation, which drives mismatch repair deficiency and the high mutation rates characteristic of these tumors. In contrast, genomically stable and chromosomal instability tumors show relatively low levels of DNA methylation, clustering in “low-methylation” groups, and may harbor other genomic alterations, such as *RHOA* mutations in GS tumors or aneuploidy and receptor tyrosine kinase amplifications in CIN tumors, but they lack the extensive methylator phenotypes seen in EBV and MSI tumors. Overall, these patterns highlight the contribution of DNA methylation to gastric cancer heterogeneity, although the exact role and definition of the CIMP in GC remain emerging and require careful interpretation.

### 3.1. Methylation as a Hallmark of the Epstein–Barr Virus and Helicobacter pylori Infections

Methylation is a hallmark of EBV-positive and MSI GC subgroups. Notably, CIMP-positive GCs arise from a different process compared to CIMP-negative tumors. Indeed, EBV-positive GCs show a high genome-wide hypermethylation and minimal demethylation out of the promoter, prevalently charged with the *CDKN2A* gene. In contrast, MSI GCs show high DNA promoter hypermethylation accompanied by demethylation out of the promoter region [36,37].

Previous studies demonstrate that EBV infection itself promotes hypermethylation of a panel of tumor suppressor genes including *MINT*, *TIMP-3*, *CDH1*, *p16*, *ACSS1*, *FAM3B*, *IHH*, and *TRABD*, and the EBV-induced hypermethylation may facilitate malignant transformation [36].

In addition, a *Helicobacter pylori* (Hp) infection is a well-established risk factor for the development of gastric cancer (GC). Maekita et al. demonstrate that a *H. pylori* infection induces aberrant DNA methylation in several gene promoters, particularly affecting genes such as *p16INK4A*, *LOX*, and *CDH1* [38,39]. Significantly, epigenetic alteration is not merely a result due to H. pylori but can be attributed to the chronic inflammation triggered by such a bacterium. In fact, reducing these effects with anti-inflammatory therapy in *H. pylori*-infected individuals can decrease polyclonal methylation, establishing the pivotal role played by chronic inflammation in such conditions. Complete eradication of *H. pylori* has been associated with a reduction in promoter methylation levels, although a residual degree of methylation often persists. Nevertheless, the overall risk of developing gastric cancer decreases following eradication, reinforcing the pathogen’s indirect significant contribution to tumorigenesis [40]. The role of both promoter hypermethylation and global hypomethylations in gastric carcinogenesis is still a topic for discussion. Many studies have found that there is a notable increase in methylation in non-neoplastic gastric mucosa affected by a *H. pylori* infection. To cite an example, a 2011 study that employed MethyLight analysis for 25 genes in 212 samples of gastric tissue discovered a clear relationship between *H. pylori* infection and hypermethylation of CpG island regions in chronic gastritis (CG) samples. It is pertinent to point out that there was no association found in samples from intestinal metaplasia (IM), gastric adenomas, or gastric cancer [40,41]. Yoshida et al. found simultaneous global hypomethylations in repetitive DNA regions (ALU, LINE-1, SATa) that occur progressively along with gastric carcinogenesis, indicating that *H. pylori* can also be a factor for hypomethylation [42]. Consequently, evaluating global DNA methylation, rather than focusing on the methylation status of individual genes, may provide a more accurate prediction of an individual’s susceptibility to developing gastric cancer. Global DNA hypomethylation is commonly observed in cancer cells and typically occurs in genomic regions distinct from those affected by promoter hypermethylation. Interestingly, genes predisposed to promoter hypermethylation often overlap with regions susceptible to hypomethylation, highlighting the complex and dynamic epigenetic landscape involved in gastric carcinogenesis. Although results across studies are sometimes divergent, accumulating evidence suggests that DNA methylation may occur early during gastric carcinogenesis and could contribute—interacting with genetic alterations, other epigenetic changes, environmental factors, and chronic inflammation—to the initial phases of tumor development. Tahara et al. (2010) [31] investigated whether promoter hypermethylation in non-neoplastic gastric mucosa could predict GC development. Analysis for the four candidate genes (*p14*, *p16*, *DAPK*, *CDH1*) disclosed a frequent promoter hypermethylation, particularly for *CDH1* and *DAPK*, in a pattern that appeared independent from patient age, gender, histology, and *H. pylori* infection [31,43]. These findings suggest the hypothesis that methylation is not merely a consequence of infection but may act as an intrinsic mechanism driving early gastric mucosal transformation, even in the absence of *H. pylori*. Moreover, the involvement of methylation in inducing intestinal metaplasia, a recognized precancerous lesion defined as a “point of no return”, further supports its role in the initial phases of gastric carcinogenesis. Nevertheless, it is important to consider that the studied cohorts are predominantly Asian, and the findings may be influenced by regional genetic and epidemiological differences. To our knowledge, only a few European studies have analyzed these epigenetic markers, but their results appear to support the conclusions outlined above [44,45,46,47].

### 3.2. MSI-Positive Gastric Cancer

Significant efforts to classify the molecular subtypes of GC have been made by both The Cancer Genome Atlas (TCGA) and the Asian Cancer Research Group (ACRG). Despite the different cohort analyzed and the disparate method applied, both classifications define MSI-positive gastric cancer as a well-defined and specific subgroup. Evidence in the literature reports a variation between 8% and 25% of MSI in gastric cancer [34,48] depending on geographical variability, heterogeneity in tumor stage distribution and the molecular analysis method applied.

Microsatellites are short, tandemly repeated DNA sequences (ranging from 1 to 6 nucleotides) distributed throughout the genome and characterized by a high mutation rate. MSI refers to a hypermutable phenotype that arises at these microsatellite regions when the DNA MMR system is deficient (dMMR). MMR machinery is a highly conserved molecular mechanism intended for repairs of mismatched nucleotides. It works thanks to several repair enzymes, named: MutL homolog 1 (MLH1), MutL homolog 3 (MLH3), MutS homolog 2 (MSH2), MutS homolog 3 (MSH3), MutS homolog 6 (MSH6), post meiotic segregation increased 1 (PMS1), and post meiotic segregation increased 2 (PMS2) [49]. MSI is most commonly associated with MLH1 deficiency by promoter hypermethylation in sporadic cases, whereas in familial forms it typically results from germline mutations in mismatch repair genes. While the correlation between *MLH1* methylation and the risk of developing gastric cancer has been explored, additional research is required [50]. Several studies highlight the presence of MSI in early stages of tumor growth and in precancerous lesions, suggesting a pivotal role in gastric carcinogenesis [51,52]. In this context, the role of *MLH1* promoter hypermethylation in the multistep process of gastric carcinogenesis remains controversial. To date, different studies have defined it as an early event in GC development, based on evidence of its detection in premalignant stages such as intestinal metaplasia and gastric adenoma [53,54,55]. Conversely, some reports have demonstrated that *MLH1* methylation can also occur as a late event in the multistep process of malignant transformation, with the subsequent silencing potentially resulting from the time-dependent accumulation of MSI [56]. Given these findings, *MLH1* promoter methylation is unlikely to represent an isolated, sporadic event; rather, it appears to occur as part of a broader epigenetic process involving hypermethylation of multiple gene promoter CpG island loci.

### 3.3. Focus on Methylation in GC Precursor Lesions

**Figure 1:**
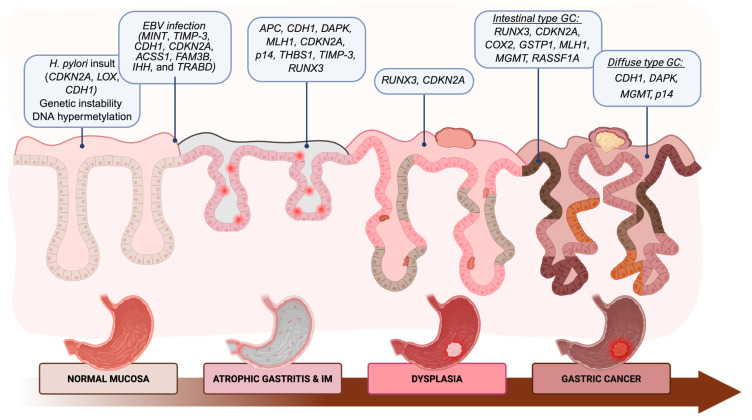
The evolution of Correa’s cascade in gastric carcinogenesis and methylated genes of interest. The progression along the Correa cascade is characterized by a gradual accumulation of epigenetic alterations, which begin already in the inflammatory/metaplastic stages (RUNX3, p16) and become consolidated in advanced lesions and carcinoma (RUNX3, CDKN2A, CDH1, MLH1), supporting the concept that DNA methylation is an early, progressive, and decisive event in gastric carcinogenesis. Created in BioRender. Ceccon, C. (2026). BioRender.com/0h2ncid.

From a diagnostic perspective, epigenetic alterations—particularly DNA methylation—represent early and widespread events in carcinogenesis, even in GCs, with relevant clinical implications [57]. The progressive transition from chronic gastritis to carcinoma is often accompanied by a stepwise increase in promoter methylation across multiple genes. To date, numerous genes have been identified as drivers of gastric cancer development, showing either hypermethylation or hypomethylation in various precursor lesions. As previously mentioned, the Correa’s cascade describes the multistep progression from normal mucosa to carcinoma [25] and further studies demonstrate how the methylation of key genes is involved in gastric carcinogenesis (Figure 1).

Of interest, the loss of *RUNX* expression by its promoter methylation has been described to occur as early as the chronic gastritis stage (8.1%), and the percentage of methylation cases increases in the later carcinogenesis stages such as IM (28.1%) and gastric adenoma (27.3%) [58].

*RUNX3* is a tumor suppressor gene on chromosome 1p36, involved, together with *RUNX1-2*, in growth, differentiation and apoptosis signals induced by the TGF-b pathway. It has also emerged as one of the gene promoters most frequently and specifically methylated in CIMP-positive forms of gastric cancer [59].

A global higher methylation rate has been observed in gastric adenocarcinoma (64%), whereas its expression in normal gastric tissue remains controversial due to a lack of consensus in the literature. Assuming that the association between DNA methylation and aging has already been demonstrated [60], the low methylation levels in normal tissue of a patient not affected by GC reported by Waki et al. [61] are more likely to reflect the advanced age of the patients studied, rather than indicating a causal relationship or a predisposing marker for gastric carcinogenesis. This interpretation is further substantiated by evidence from Kim et al., which demonstrates an increase in methylation with age in a similar cohort of patients not affected by GC. Of note, *RUNX3* promoter methylation is cancer-related, since its methylation has not been detected in other non-neoplastic tissue (chronic hepatitis, colon and prostate) [58]. According to the literature, the *CDKN2A* (*p16*) gene may also be involved in gastric precancerous lesion malignant transformation since it was found to be increasingly methylated from IM (7%), adenomas (18%), to carcinoma (44%) [62,63]. *CDKN2A* is involved in the cell cycle G1/S phase checkpoint, and its downregulation leads to uncontrolled proliferation. Of note, to decipher the role of *p16* methylation in non-neoplastic gastric mucosa, a 2002 study by Waki et al. compared the methylation of this gene among non-neoplastic cells of different organs from autopsy and the normal counterpart in the stomach matched with the respective tumor. They found higher methylation in non-neoplastic cells of patients older than 45, and no methylation in 22 year-old patients or younger. Interestingly, this seems not to be an age-dependent methylation, because in the matched normal mucosa and adenocarcinoma from patients with GC, methylation was frequently observed in both neoplastic and corresponding non-neoplastic gastric epithelia, making *p16* a distinctive marker suggestive of neoplastic potential transformation [61]. Furthermore, Osawa et al. also underline an association between the marked decrease in *p16* expression, related to the methylation process, and the development of EBV-associated gastric carcinoma (EBVaGC) [64]. Of note, the *p16* gene is predominantly methylated in the intestinal type of GC; unlike this, *CDH1* is hypermethylated in diffuse gastric cancer (DGC) [65]. This evidence underlies a possible correlation between epigenetic changes and cancer morphology.

*CDH1* is a well-studied gene encoding the cadherin-E protein, a calcium-dependent protein involved in cell adhesion and intrusion suppression [66]. Reduced expression of *CDH1* promotes cancer invasion and contributes to the metastasizing process. Moreover, the cooperation of DNA hypermethylation and the A allele of the −160 C → A polymorphism affecting *CDH1* has been described to increase the risk of developing GC. This evidence reinforces the well-established idea of the synergic activity of molecular and epigenetic alteration in promoting cancer development [67,68].

A recent case–control study emphasizes the previous findings identifying differences in the methylation status of *CDH1* between patients with diffuse gastric cancer and healthy controls. Furthermore, a statistically significant association was observed between the poorly differentiated histological subtype and advanced stages of gastric carcinogenesis. Given that poorly differentiated tumors are typically associated with more advanced stages of disease, these findings further support the hypothesis that *CDH1* methylation may serve not only as a diagnostic marker but also as a potential prognostic indicator in diffuse gastric cancer [46,69]. Supporting this hypothesis, a 2013 study had already reported concurrent *CDH1* promoter methylation in both gastric tumor tissue and adjacent non-neoplastic mucosa. This epigenetic alteration likely represents an early event in gastric carcinogenesis that precedes tumor development and progresses in severity with advancing disease stage [70]. Of interest, a recent organoid-based study outlined increased methylation levels in specific CpG island sites in IM compared to normal tissue, suggesting that dysregulation of the putative signaling pathway occurs before malignant transformation. This theory was further refined with additional information; while methylation signatures change between IM and normal tissue, the hypermethylation of specific genes does not change between IM and GC. This additionally not only supports the concept that IM is an irreversible lesion, but also outlines that aberrant methylation starting in IM continues in GC, eventually promoting deregulated expression [71]. Despite several studies supporting this theory, a 2021 study seems to contrast this evidence. Indeed, Sugimoto et al. did not observe *MLH1* methylation in isolated non-neoplastic samples obtained from the surrounding mucosa of gastric cancer with a MSI phenotype. To our knowledge, this is the only study using isolated glands without interstitial cells, which may explain the discrepancy with previous results [51]. Notwithstanding these discrepancies, mainly due to cases selection, Yoda et al.’s study combined the epigenetic and genetic alterations in 50 GCs, observing that the *MLH1* gene, together with *CDH1* and *CDKN2A*, were more frequently inactivated by epigenetic alterations rather than genetic mutations [72]. The role of *MLH1* in preneoplastic GC setting still remains unclear. The correlation between the lack of MLH1 protein expression and high MSI (MSI-H) has been well established since 1999, and indicates the pivotal role of the *MLH1* gene for MSI-H status in GC [73]. This strong correlation was also observed in preneoplastic gastric lesions and early gastric adenocarcinoma. On the other hand, on this topic, contrasting results have been obtained analyzing non-neoplastic surrounding mucosa. Indeed, the *MLH1* promoter methylation was found in 6.3% of mucosa with IM [53] and up to 71% of non-neoplastic mucosa [74], but some studies did not reveal any methylation in any of the non-neoplastic mucosa, including IM [51,55].

To date, our knowledge of methylation in the preneoplastic setting is still scarce and controversial, mainly due to limitations in analytical methods and case selection, which introduce bias and lead to discrepancies in the final conclusions. The need for more in-depth and standardized studies in case series could be the key to more reliable results.

## 4. Experimental Strategies Suitable for Diagnostic Practice

In the diagnostic setting, the main goals to reach are sensitivity, specificity, analytical time-to-results and reproducibility of the results. It is challenging to find a test that satisfies all these requirements.

The available approaches can be divided into three categories: (1) bisulfite conversion-based methods; (2) restriction enzyme-based assay; and (3) the affinity enrichment-based approach. Nowadays, bisulfite conversion methods are mostly used, but other assays can be considered according to the nature of biological problems under investigation, laboratory facilities and technology, economical possibilities, and the needed resolution (Table 1).

### 4.1. Bisulfite Conversion and Method Based on Converted DNA

The bisulfite conversion approach was described for the first time in 1979 by Hayatsu and Shiragami [75], but only at the end of the ’80 were the pioneer studies which applied the assay to solid tumor research and further refined the protocols published [76,77,78,79].

The method is based on a chemical modification of the cytosines residue by sodium bisulfite [76]. The main limits of this method are the fragmentation of DNA due to harsh reaction conditions, incomplete conversion which generates false-positive results and reduction in complexity of DNA from 4 to 3 bases [80]; indeed, the low amount of DNA input and the degraded DNA (such as the FFPE tissues) are not a limitation anymore because of the newest sensible available kits [81].

The converted DNA can be analyzed through different PCR-based methods: (i) methylation-specific PCR (MSP) [82,83]; (ii) methylation-sensitive high-resolution technology (MS-HRM) [81,84]; (iii) bisulfite sequencing PCR [76]; (iv) bisulfite pyrosequencing [85,86,87]; (v) droplet digital PCR (ddPCR) [88,89,90]; (vi) Epityper by Agena Bioscience (Sequenom, San Diego, CA) [91].

### 4.2. Methylation-Specific Restriction Enzymes-Based Approaches (MSRE)

MSRE-based approaches profile DNA methylation by exploiting restriction enzymes (mainly HpaII, MspI) sensitive to cytosine methylation. After enzymatic digestion, methylation status is assessed using PCR/qPCR, ddPCR, microarrays, or NGS by comparing signals from digested versus undigested DNA to estimate methylation levels. However, MSRE-based methods are rarely used in routine clinical practice due to restriction site limitations, dependence on efficient digestion, lower sensitivity and quantification accuracy compared with bisulfite-based methods, and poor inter-laboratory standardization [92,93].

### 4.3. Affinity Enrichment-Based Approaches

Affinity enrichment-based approaches profile DNA methylation by selectively capturing methylated DNA fragments through affinity interactions. Genomic DNA is fragmented and methylated sequences are enriched using methods such as methylated DNA immunoprecipitation (MeDIP) or methyl-CpG binding domain (MBD) protein-based assays [94]. The enriched DNA can then be analyzed by PCR-based techniques (qPCR, ddPCR), microarrays, or next-generation sequencing for genome-wide profiling [81,95]. These methods do not require bisulfite conversion, preserve DNA integrity, are suitable for large-scale analyses, and are more cost-effective than whole-genome bisulfite sequencing [94].

Pre-designed kits have simplified restriction enzymes and affinity-based methylation assays, making them more user-friendly and compatible with a wide range of analytical methods and target panels. These ready-to-use and often automatable kits support applications ranging from whole-genome methylation profiling on high-throughput platforms (e.g., Illumina NovaSeq, Illumina, San Diego, CA, USA) to more robust analyses using pre-designed or custom NGS target enrichment panels. While large panels enable simultaneous assessment of multiple genomic regions, they also increase data complexity, potentially prolonging reporting times and requiring greater analytical expertise and computational resources.

### 4.4. Methodological Variability and Its Impact on Biomarker Reproducibility

Methodological variability represents a significant challenge in translating DNA methylation research into clinical practice, as it directly introduces bias and leads to discrepancies in final conclusions regarding biomarker efficacy. The lack of standardization across different assay platforms, sample types, and analytical thresholds remains a primary hurdle to achieving the reproducibility required for clinical utility. Several technical factors contribute to inconsistent results; first of all, different analytical techniques offer varying levels of reliability. For instance, MSP is noted for having a less standardized output, making it less consistent compared to other techniques [83]. Conversely, bisulfite pyrosequencing is considered the “gold standard”, specifically because of its high degree of result standardization and reproducibility. Of note, we must consider that all the bisulfite-based conversion methods can suffer from DNA fragmentation and incomplete conversion, which generates false-positive results [87]. Similarly, the MSRE method faces significant challenges with inter-laboratory standardization because its quantification is less reliable and sensitive than bisulfite-based methods [93]. Again, the affinity enrichment-based approaches require complex computational normalization for CpG content, a process that is not as standardized as other methodologies [96]. Currently, only highly precise techniques like Droplet Digital PCR (ddPCR), which allows for absolute quantification and high signal-to-noise ratios, are deemed suitable for the rigorous demands of detecting rare targets in diverse sample types like liquid biopsies [90,97].

**Table 1:** Comparison of experimental approaches for DNA methylation analysis.

Method	Bisulfite Conversion-Based							Restriction Enzyme-Based	Affinity Enrichment-Based	
Assay	Methylation Specific PCR (MSP)		Methylation Sensitive High-Resolution Technology (MS-HRM)	Bisulfite Sequencing PCR	Bisulfite Pirosequencing	Droplet Digital PCR (ddPCR)	Epityper		MeDIP	MDB
First Appearence	2000 [83]	2004 [84]	2008 [85]	1992 [77]	2003 [86]	1999–2005 [89]	2005 [92]	2005 [93,94]	2005 [95]	1994 [95]
	MethyLight	MethylQuant								
**Principle of** **the method**	Fluorescence-based real-time PCR (TaqMan®). Two sets of primer amplify specifically methylated and unmethylated CpG regions.	Fluorescence-based real-time PCR (SYBR Green). Two sets of primer amplify specifically methylated and unmethylated CpG regions.	PCR-based method. Two sets of primer recognize methylated and unmethylated DNA. Melting temperature evaluation.	Single or semi-nested PCR using CpG-independent primers amplifies both methylated and unmethylated alleles, followed by gel electrophoresis and Sanger sequencing to resolve regional methylation patterns at single-base resolution.	Bisulfite pyrosequencing is a sequencing-by-synthesis method that quantitatively measures DNA methylation at individual CpG sites by detecting light signals generated during nucleotide incorporation.	DNA partitions into thousands of droplets to achieve absolute and highly sensitive quantification of methylated targets by counting positive versus negative droplets.	A region-specific, quantitative DNA methylation method combining bisulfite PCR with MALDI-TOF mass spectrometry to distinguish methylated from unmethylated cytosines based on fragment mass, enabling percentage-based quantification at CpG sites or CpG units.	Methylation-sensitive restriction enzymes to selectively digest unmethylated DNA, followed by PCR-, array-, or sequencing-based analysis. Comparison of digested and undigested samples allows estimation of regional CpG methylation levels.	An affinity-based method in which fragmented genomic DNA is enriched using antibodies specific for 5-methylcytosine, followed by PCR-, microarray-, or NGS-based analysis to profile methylated regions genome-wide without bisulfite conversion.	These approaches use methyl-CpG binding domain proteins to selectively capture methylated DNA fragments, which are then analyzed by PCR, microarrays, or NGS, enabling cost-effective genome-wide methylation profiling, with bias toward CpG-dense regions.
**Quantitative**	Precise quantification	Precise quantification	Semi-quantitative	Approximate/qualitative	Precise quantification	Precise quantification	Precise quantification	Approximate/qualitative	Depending on the analysis method chosen	Depending on the analysis method chosen
**Sensitivity**	High	Medium	High	Low	Medium	High	Medium	Medium	Medium	Medium
**Specificity**	High	Medium	Medium	High	High	High	High	High	High	Medium
**Resolution**	Low	Medium	Medium	High	High	High	High	Low	Medium	Medium
**Advantages**	Highly accurate, ready-to-use assay, flexible selection of interest region. [82]	Economical. [82]	Fast, easy and ready-to-use, detects heterogeneous methylation, allows estimation of relative DNA methylation percentages. [82]	Single based resolution for specific regions. [77]	Standardized and reproducible, good cost-to-quality ratio, simultaneous quantification of multiple CpG sites. [88]	Fast, absolute quantification of DNA, ble to detect small methylation differences, easy to interpret, suitable for different sample type. [90,91]	Fast, region-specific, Region-specific, high-resolution, large numbers of samples can be analyzed. [92]	Fast, easy of use, can be combined with PCR, qPCR, ddPCR, microarrays, or NGS, avoids some of the problems inherent in bisulfite conversion, detects heterogeneous methylation. [93,94]	No bisulfite conversion, suitable for genome-wide methylation profiling, cost-effective compared to whole-genome bisulfite sequencing. [82,96]	Economical, no bisulfite conversion, efficient for capturing highly methylated regions, genome-wide applications possible with PCR, microarray, or NGS. [82,96]
**Limitations**	Costly, cannot detect heterogeneous methylatio, lack of standardization, limited number of CpG sites at once. [82]	Unsuitable for the analysis of heterogeneous pathological samples, lack of standardization. [82]	Limited to regions with multiple CpGs; cannot analyze single CpG sites. [82]	Sequencing can be noisy, time-consuming, costly. [77]	Limited read length (~50–60 bp per amplicon), restricts the number of CpG sites analyzed per assay. [88]	Laborious primer design, especially with dense CpG regions, FFPE and liquid biopsy samples may require triplicates for reliability. [90,91]	Expertise in mass spectrometry, SNPs in target regions can complicate interpretation, high cost for large-scale or high-throughput studies. [92]	Limited to CpG sites present in restriction enzyme recognition sequences, requires complete and efficient enzymatic digestion, quantification is less reliable and less sensitive than bisulfite-based methods, difficult to standardize across laboratories. [93,94]	Costly, CpG-poor regions may be underrepresented, requires computational normalization for CpG content bias, not standardized across laboratories. [82,96]	CpG-poor regions may be underrepresented, requires computational normalization for CpG content bias, not standardized across laboratories. [82,96]

The techniques used in the analysis of DNA methylation can be broadly classified into bisulfite conversion-based techniques, restriction enzyme-based techniques, and affinity enrichment-based techniques. Bisulfite conversion-based techniques include MSP, MethyLight, bisulfite sequencing, pyrosequencing, and digital PCR. These techniques are highly accurate and allow for single-nucleotide resolution, with the latter being the most preferred for quantitative purposes. However, these techniques are limited in throughput and are often challenged by heterogeneous samples. On the other hand, restriction enzyme-based techniques are simple and do not alter the native DNA but are limited to restriction site-specific recognition and are less sensitive. Affinity enrichment techniques, including MeDIP and MBD, are useful for genome-wide studies but are biased towards CpG-rich regions. Next-generation sequencing (NGS) is often used in combination with the aforementioned techniques to allow for genome-wide studies with high throughput. This provides a wide range of molecular information at the expense of complexity and longer reporting times. The resolution, specificity, quantitative performance, sensitivity, accuracy, and reproducibility of the assays are expressed on a scale from “Low” to “High”. “Low” indicates low resolution, limited specificity, unreliable quantification, low sensitivity, low accuracy, and poor reproducibility, whereas “High” denotes single-base resolution, high specificity, reliable quantitative performance, high sensitivity, high accuracy, and strong reproducibility.

## 5. GC Clinical Management

The significant inter- and intratumoral heterogeneity of GCs are a major factor contributing to the poor clinical outcomes associated with this malignancy and a deeper understanding of tumor biology is essential to optimize therapeutic efficacy [98]. While radical surgery remains the cornerstone of treatment for localized disease, multimodal strategies—including perioperative chemotherapy and chemoimmunotherapy or adjuvant chemo—have been implemented to reduce recurrence risk and extend overall survival [99,100].

The perioperative landscape for resectable GC has been recently redefined by the MATTERHORN phase III trial, which integrated durvalumab (anti-PD-L1) with the FLOT (5-fluorouracil, oxaliplatin and docetaxel) backbone. This combination significantly improved Event-Free Survival (EFS), primary endpoint, and increased the pathological Complete Response (pCR) rate from 7% to 19% while long-term survival data are still maturing. This synergistic approach not only achieves immediate tumor regression but is designed to foster a durable systemic immune response, potentially mitigating long-term recurrence risk and establishing perioperative chemo–immunotherapy as a new therapeutic benchmark [101]. In the metastatic setting, systemic therapy is the cornerstone of treatment. Current standards prioritize doublet chemotherapy—typically combining fluoropyrimidine with a platinum agent—due to a favorable balance between efficacy and toxicity. Notably, oxaliplatin has largely superseded cisplatin, particularly for older or more fragile patients, owing to its superior tolerability [102]. However, the most significant shift in first-line care is the mandatory use of molecular profiling.

For HER2-negative tumors, the integration of ICIs—most notably nivolumab, pembrolizumab, and tislelizumab—into first-line chemotherapy has changed the standard of care [101,103,104]. The most successful targeted treatment for GC patients is HER2, the overexpression of which—primarily driven by gene amplification—is present in approximately 15% of cases [105]. Since the landmark ToGA trial, adding trastuzumab to fluoropyrimidine/platinum chemotherapy has been the global first-line standard. Recently, this paradigm has been further advanced by the KEYNOTE-811 phase III trial, which demonstrated that integrating pembrolizumab into the trastuzumab–chemotherapy backbone significantly improves the objective response rate (ORR) and progression-free survival (PFS), particularly in tumors with PD-L1 CPS 1. This triple-combination therapy now represents the new benchmark for HER2-positive advanced gastric adenocarcinoma [106]. A major milestone in the molecular management of HER2-negative GC is the targeting of Claudin 18.2 (CLDN18.2). Based on the SPOTLIGHT and GLOW phase III trials, the monoclonal antibody zolbetuximab has established a new standard of care. When added to chemotherapy (mFOLFOX6 or CAPOX), zolbetuximab significantly improves PFS and OS in patients with CLDN18.2-positive tumors [107,108].

Nevertheless, further outcome improvement is warranted and, moreover, there is a critical need of early cancer detection and more robust recurrence-prevention strategies. In this regard, liquid biopsy and the analysis of circulating molecules offer promising potential for predicting disease relapse [100]. Despite these advances, clinical benefit remains limited and resistance to ICIs represents a formidable challenge; therefore, a comprehensive investigation of epigenetics regulation, metabolic pathways, the microbiome, and the tumor immune microenvironment is required to elucidate the complex mechanisms underlying immune modulation and therapeutic resistance [109,110].

### DNA Methyltransferase Inhibitors Therapeutic Opportunities

From a translational perspective, the integration of epigenetic modulation into the therapeutic armamentarium could be informative on pharmacological resistance and tumor sensitivity to conventional and targeted interventions. However, epigenetic-targeted strategies remain largely exploratory in GC; although DNA methylation patterns influence gene expression programs and may interact with chemotherapy and other targeted therapies, their impact on patients’ outcomes still requires robust clinical validation [111]. In the clinical management of GC, the epigenetic landscape does not merely reflect tumor progression but serves as a decisive factor in determining the success or failure of conventional chemotherapy. The failure of cytotoxic regimens is frequently driven by the silencing of genes essential for metabolic drug activation or the initiation of programmed cell death. By silencing key regulatory pathways, hypermethylation can create a resistant environment; though in select cases, it can paradoxically render the tumor more vulnerable to intervention.

Specifically, hypermethylation of key genes involved in cell-cycle regulation, DNA repair, and apoptosis—such as *TFAP2E*, *TMS1*, *ABCB1* and *SRBC*—has been directly correlated with resistance to radiotherapy and standard chemotherapeutic agents like 5-FU, cisplatin and oxaliplatin, whereas distinct profiles such as *CDKN2A* hypermethylation or *ADGRL2* hypomethylation may predict chemosensitivity [112,113,114].

The rationale lies in the lack of this cell-cycle inhibitor, which results in unregulated proliferation; this hyper-proliferative state makes the cell particularly susceptible to agents that target DNA synthesis and integrity. Furthermore, the epigenetic modulation of *DCTPP1* (dCTP pyrophosphatase 1) has emerged as a critical regulator of the intracellular nucleotide pool. By disrupting pyrimidine metabolism, the specific methylation status of *DCTPP1* can sensitize gastric cancer cells to 5-FU, turning a metabolic pathway into a therapeutic vulnerability [115]. PD-L1 promoter hypermethylation has been observed in patients following anti-PD-L1 therapy, suggesting a mechanism of acquired resistance [116]. Conversely, PD-L2 hypermethylation correlates with an EBV status, MSI, and high mutational load, potentially serving as a predictive biomarker for immunotherapy response [117]. To counteract these epigenetic alterations, DNA methyltransferase inhibitors (DNMTis), including nucleoside analogs (azacitidine, decitabine) and novel non-nucleoside compounds, are being actively investigated. Particularly, nucleoside analogs act as cytosine mimics that incorporate into the genome or transcriptome during cellular replication, leading to the depletion of DNMT1 and other methyltransferases. Unlike azacytidine, decitabine (DAC) is directly incorporated into DNA, where it inhibits DNA methylation and promotes reactivation of previously silenced genes. This effect is particularly important for genes involved in cellular differentiation, as decitabine induces a more differentiated cellular state. About gastric cancer, a recent in vitro study demonstrated a significant reduction in cell proliferation, migration, and invasion without affecting cell viability after DAC treatment. Additionally, transcriptomic analysis revealed that DAC-treated PDGC cells upregulated multiple immune-related genes, highlighting the therapeutic potential of this strategy [36,118].

Zebularine is a younger agent in the class which acts as a stable cytidine analog that reduces DNMT protein expression and reactivates genes silenced by hypermethylation, offering a potentially lower-toxicity profile for long-term administration [36,119]. Although clinical evidence suggests that DNMTis alone may be insufficient to fully reactivate silenced genes, their combination with traditional chemotherapy has demonstrated a promising synergistic effect in restoring chemosensitivity; for instance, neoadjuvant priming with 5-azacitidine prior to EOX (epirubicin, oxaliplatin and capecitabine) therapy has been shown to induce significant hypomethylation of tumor-related loci and improve clinical response. The clinical value of these inhibitors extends beyond single-agent efficacy, offering a potent strategy to overcome therapeutic resistance. By restoring the expression of genes involved in apoptosis and drug sensitivity, DNMTis can “prime” the tumor, making it more vulnerable to conventional chemotherapy or immunotherapy [120,121]. Furthermore, epigenetic profiling is informative for post-treatment surveillance, as the hypermethylation of miR-34b/c, *SFRP2*, and *DKK2* serves as a strong predictor for the development of disease recurrence after endoscopic resection. Ultimately, the complex interplay between methylation patterns, infectious agents (EBV, *H. pylori*), and immune checkpoints like PD-L1 underscores the necessity of integrating epigenetic biomarkers into clinical practice to refine prognostic accuracy and develop personalized therapeutic strategies [122]. Indeed, *H. pylori* infection impacts DNA hypermethylation in the promoter region of specific genes, which remains even after bacterial eradication, being the bridge to the occurrence of GC [123,124,125].

Possible future opportunities regard Nicotinamide N-methyltransferase (NNMT), which functions as a critical cytosolic enzyme that orchestrates the transfer of a methyl group from S-adenosylmethionine (SAM) to nicotinamide (NAM), yielding S-adenosylhomocysteine (SAH) and 1-methylnicotinamide (1-MNAM). By sequestering SAM, NNMT leads to a global decrease in histone methylation, and therefore, to the cell’s epigenetic landscape reprogramming and pro-tumorigenic gene expression activation [126].

In the context of GC, NNMT was found to be increased in patients’ exosomes compared with healthy donors [127]. Considering pathophysiology, NNMT is both necessary and sufficient to drive the phenotypic transition of quiescent fibroblasts into activated, “unfavorable” CAFs. These NNMT-high CAFs facilitate disease progression by secreting oncogenic factors, such as LOXL2, which promote extracellular matrix remodeling, enhanced tensional homeostasis, and a permissive environment for EMT. Indeed, stromal NNMT expression has emerged as a superior prognostic biomarker compared to epithelial expression, associated with diminished patient survival [127,128].

Considering clinical utility, NNMT levels were found to correlate directly with the advanced TNM stage of the tumor [128] and it was significantly increased in exosomes isolated from GC patients with peritoneal metastases compared to others [127].

The detection of DNA methylation in cell-free DNA, as well as gastric juice and stool, has been evaluated as a testing material. Studies have reported gene-specific sensitivities ranging from 47% to 94% and specificities from 50% to 100% in blood samples; 23–67% sensitivity and 79–98% specificity in fecal samples; and 65–95% sensitivity and 83–100% specificity in gastric juice [129].

Among those investigated, *SFRP2* and *RPRM* showed significantly higher prevalence in GC patients’ serum compared to patients with chronic gastritis or healthy subjects. Evaluating the biomarkers together in a dual-genes panel granted 57.58% of sensitivity and 96.25% of specificity for malignancy detection [130]. Additionally, in *H. pylori*-negative patients, *TWIST1* and *GCMI* have been reported to be significantly higher in patients with GC compared to controls [131]. Overall, researchers are seeking reliable biomarkers to guide diagnosis and treatment; however, conclusive data regarding preferred candidates or those with robust clinical backing remain elusive. This lack of consensus is driven by a fragmented landscape of findings and limited scale of existing studies, derived from small, often single-center patient cohorts ranging from only 50 to 100 participants [129]. Consequently, despite significant efforts, achieving rigorous reproducibility and cross-platform validation in larger, more diverse cohorts remains a major hurdle. Addressing these gaps is an essential prerequisite before these tools can undergo the formal evaluation required for routine clinical implementation and personalized patient care [129,130].To this point, while it is premature for interventional clinical trials, ongoing observational studies are currently evaluating various biomarkers. Among others, researchers are focused on investigating fecal methylation (NCT07312500) [125], combined assays for cell-free DNA (cfDNA) methylation alongside blood-based biomarkers (NCT05347524) [132], and the development of a comprehensive methylation signature panel for gastric cancer patients(NCT06440018) [133].

## 6. Conclusions and Future Directions

Given the rapid evolution of GC epigenetic research over recent decades, it is imperative to further investigate these mechanistic insights. However, integration into current clinical framework is not warranted with data available up to date. Key limitations also need to be addressed, such as potential differences in marker performance due to tumoral heterogeneity, histological subtype (intestinal vs. diffuse), and anatomic location.

Moreover, the diversity of sample types—such as tissue biopsies, cell-free DNA (cfDNA), gastric juice, and fecal samples—adds significant complexity to the landscape of biomarker identification and comparison. This overall heterogeneity underscores the need for methodological standardization, to ensure reproducibility and to enable reliable cross-study comparisons and advance clinical translation. The impact of this variability is also evident in the contradictory findings in the timing of methylation events (early vs. late), such as the MLH1 promoter. Indeed, the transition from preclinical promise to clinical utility requires additional effort focused on translational applications. Following the identification of biomarkers, panels require larger clinical adoption.

## Data Availability

No new data were created or analyzed in this study. Data sharing is not applicable to this article.
